# Supplementary material for: Dragondrop: a novel passive mechanism for aerial righting in the dragonfly
Source: Proc Biol Sci. 2021 Feb 10;288(1944):20202676. doi: 10.1098/rspb.2020.2676 (PMC7893233; doi:10.1098/rspb.2020.2676)
Supplement: Supplementary Materials [file rspb20202676supp2.pdf]

DragonDrop: A novel passive mechanism for aerial righting in the dragonfly

Proceedings of the Royal Society B

Samuel T. Fabian, Rui Zhou, and Huai-Ti Lin

DOI 10.1098/rspb.2020.2676

Supplementary Materials and Methods

The dragonflies

18 male common darters (*Sympetrum striolatum*) were captured around the SE of the UK. Males were used as they were more abundant, and we chose animals that weighed > 200 mg. All animals were used within 3 days of capture. We occasionally kept animals in the fridge at 8°C to increase longevity.

Data analyses

Labelling of trajectories was conducted in Qualisys Track Manager software and exported. All further analyses were conducted in MATLAB using custom written scripts. Stereoscopic remapping of the animal markers to the anatomical frame was done via the DLTdv8 app [1]. All reported statistics are given as means ± standard deviation. All significance statements are given after Bonferroni correction of our probability threshold ($p < 0.05$) for the number of comparisons.

The release apparatus

A small piece of 0.5 mm thick ferrous magnet (<10 mg) was glued below the hindlegs close to the animal's centre-of-mass. Our magnetic platform could release an inverted dragonfly by pulling a strong magnet away from the top side of the upper side (Figure S1). Animals began to fall 167 ± 8 ms after the trigger pulse was received. To release a dragonfly dorsal-side-up, we built a retractable platform which pulled away quickly under a perched dragonfly (Figure S1). The retraction generated air currents and slightly accelerated the dragonfly at the initial 30 ms, which we marked in all data plots for reference.

Insect scale motion capture and behavioural arena

All dragonfly behaviour was recorded within our custom flight arena (Figure S1). Animals were dropped centrally, 2 m from the arena floor. A panoramic print of one of our field sites was hung to provide visual texture. Lighting arrangement was done after [2]. Reference videos were collected using a SONY RX0 camera shooting at 240 FPS.

We used a Qualisys Miqus M5 camera system and custom markers to capture the kinematics of the animal's trajectory following [2]. We produced insect-scale markers by press-fitting retro-reflective adhesive over 0.8 mm diameter glass spheres. Each marker weighed approximately 1 mg (Figure S2). The body frame marking can be referred to [2]. Two head markers were attached the interocular-axis between the postclypeus and frons via a 4mm long carbon rod. Finally, a marker was affixed to the 7th abdominal segment. We achieved tracking accuracy within 150 µm. We numerically reconstructed the

neck- and abdomen-joints as needed based on the relative marker movements. Body marker frame kinematics were mapped to the dragonfly's biomechanics body reference frame. This was done via a 3D digitization of the markers' positions relative to the dragonfly's anatomical features. Details can be referred to [2]. The body axis elevation was set by aligning to the average velocity vector during level flight (25±13° below the wing plane).

Dragonfly centre of mass measurement

We calculated the centre of mass of dragonflies that had been anaesthetised on ice. Dragonflies were first weighed. We then rested a known-length and known-weight beam on two pivots (upturned razor blades) of equal height. The pivot had equidistant 1 cm marks along its length. One pivot was fixed, while the other rested on a microbalance scale. We recorded the weight of the pivoting bar. We then hung the immobile dragonfly from the beam at various arbitrary positions. At each position, we noted the recorded mass on the scale, and took a low-parallax image (100 mm focal length, 35 mm equivalent) of the dragonfly on the beam. We then used a custom written MATLAB script to digitise the bar length in 2D. We took the mass difference between the unladen beam and beam + dragonfly. We then multiplied the beam length by the measured mass difference, and subsequently divided by measured dragonfly mass. This gave the distance along the bar of the dragonfly centre of mass, which was vertically extrapolated onto the dragonfly. We consistently found that the dragonfly's centre of mass in all cases was within 3 mm of the thorax-abdomen joint.

Inverse dynamic modelling

A numerical inverse dynamic model was constructed in SimScape (MATLAB 2020a) (Figure S6). A dragonfly model was derived from a collaborator's contribution and modified to match the average body segment dimension and density of recently dead *Sympetrum striolatum*. The average weight of each body segment was determined by weighing these segments from 6 recently dead dragonflies. This allowed segment specific density estimation. For simplicity, the inertial contribution from the wings was modelled as point mass with measured wing weight and centre of mass (0.0008g per wing, 16mm from the wing joints). A cartesian joint and a gimbal joint were connected to the model centre of mass to replicate the manoeuvre. To minimise the effect of force on torque simulation, the model estimated the global forces and torques in each frame using a fixed step ode1 backward Euler solver in a zero-gravity environment. We then added a constant gravitational force(-9.80665N/kg) onto the estimated global force remapped the outputs into body reference frame to compute the body forces and torques. The model details are shown in Figure S6.

Desktop wind tunnel

We built a custom 3D printed low-speed vertical wind tunnel (9 x 9 cm outlet, printed in PLA) connected to an 65mm electric ducted fan (six blades; 4000Kv motor) for testing the torque generated from animal wing positioning. The outlet speed was manually tuned to 2.4 $m.s^{-1}$ (roughly the terminal

velocity of a freely falling dragonfly) using a digital anemometer (BTMeter BT-100, ± 0.1 $m.s^{-1}$). We attached the animal on a pivot-bar that ran across the wind tunnel outlet (Figure S7). The pivot bar extended outside of the outlet and interfaced with a cantilever transducer (SMD S100) to provide torque measurement. In each measurement we collected 10 seconds of baseline data without airflow and compared its mean to five seconds of constant airflow. The cantilever transducer was calibrated separately using known weights.

References:

1. Hedrick TL. 2008. Software techniques for two-and three-dimensional kinematic measurements of biological and biomimetic systems. *Bioinspiration & biomimetics*, 3(3), p.034001.
2. Mischiati M, Lin H-T, Herold P, Imler E, Olberg R, Leonardo A. 2015. Internal models direct dragonfly interception steering. *Nature*, 517(7534), pp.333-338.

DOI 10.1098/rspb.2020.2676

Supplementary Figures:

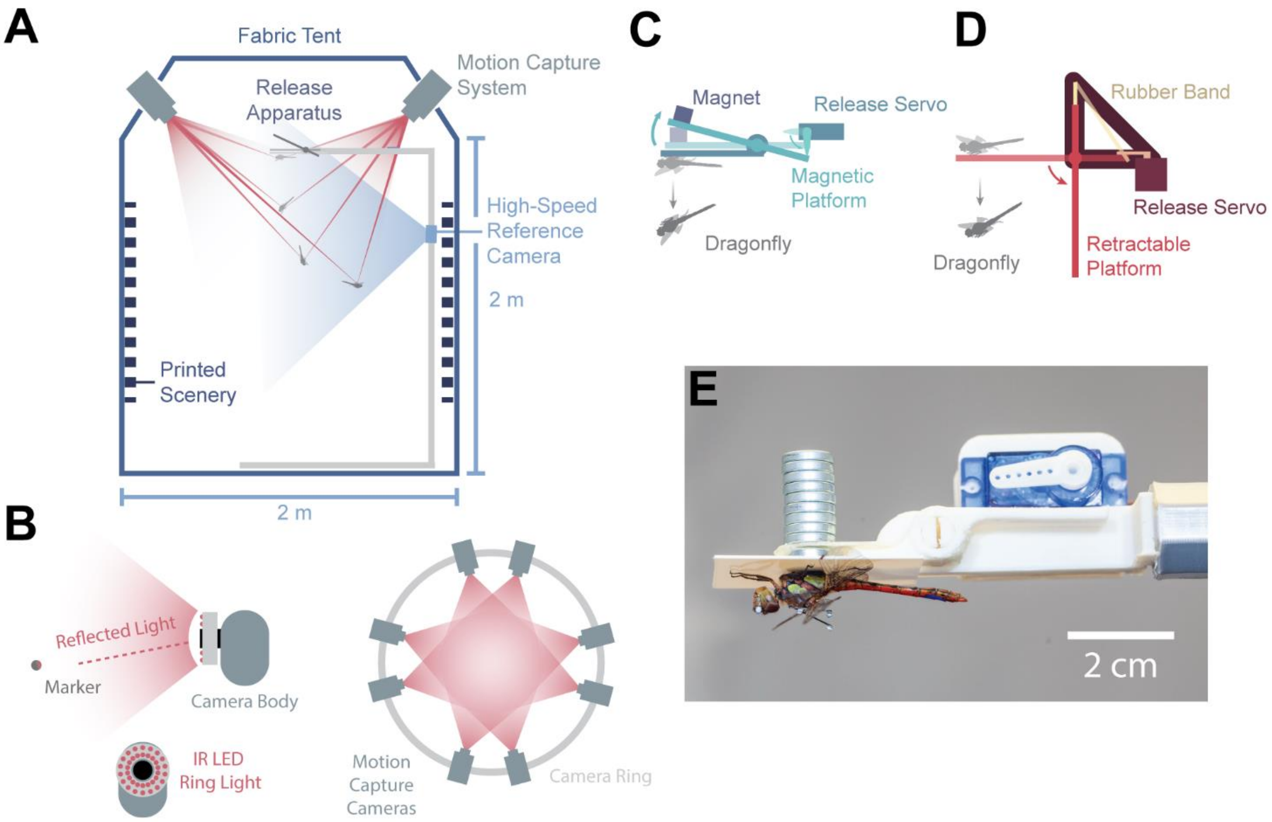

Supplementary Figure 1: Motion capture and the release mechanism

(A) A schematic drawing of the behavioural tent in which experiments were conducted. *(B)* A schematic of the principles of motion capture and arrangement of cameras in the behavioural arena. *(C)* The inverted drop magnetic platform. *(D)* The dorsal-side-up drop retractable platform. *(E)* A photo of a marked dragonfly on the magnetic platform.

DOI 10.1098/rspb.2020.2676

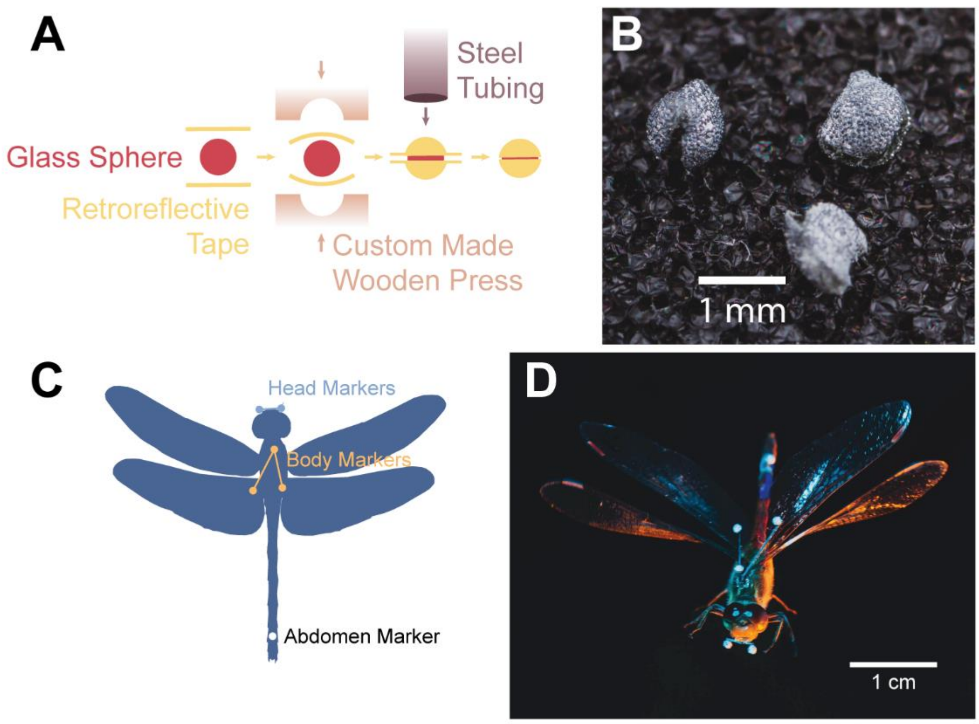

Supplementary Figure 2: The retroreflective markers and their fabrication

(A) A schematic representation of our new motion capture marker manufacture process. (*B*) Macro photograph of custom markers which will go through further screening. (*C*) Schematic for the 5-marker arrangement applied to the dragonflies. (*D*) A marked common darter (*Sympetrum striolatum*) dragonfly in mid-fall.

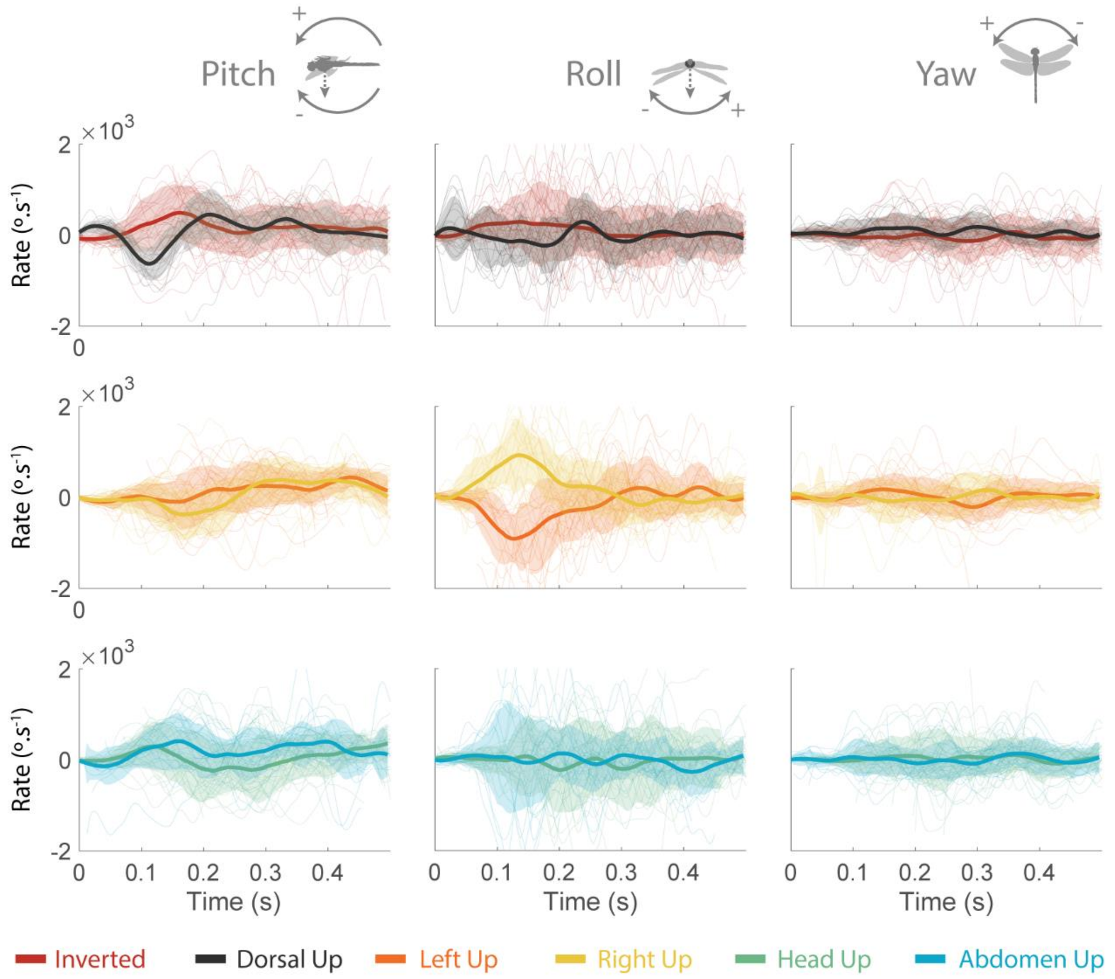

Supplementary Figure 3: Pitch, Roll, and Yaw rates by initial orientation

The angular rotation rates of the dragonflies' body are given in pitch (left), roll (middle), and yaw (right). Population means are given in bold, colour coordinated to the initial condition. Shaded area shows ± STD.

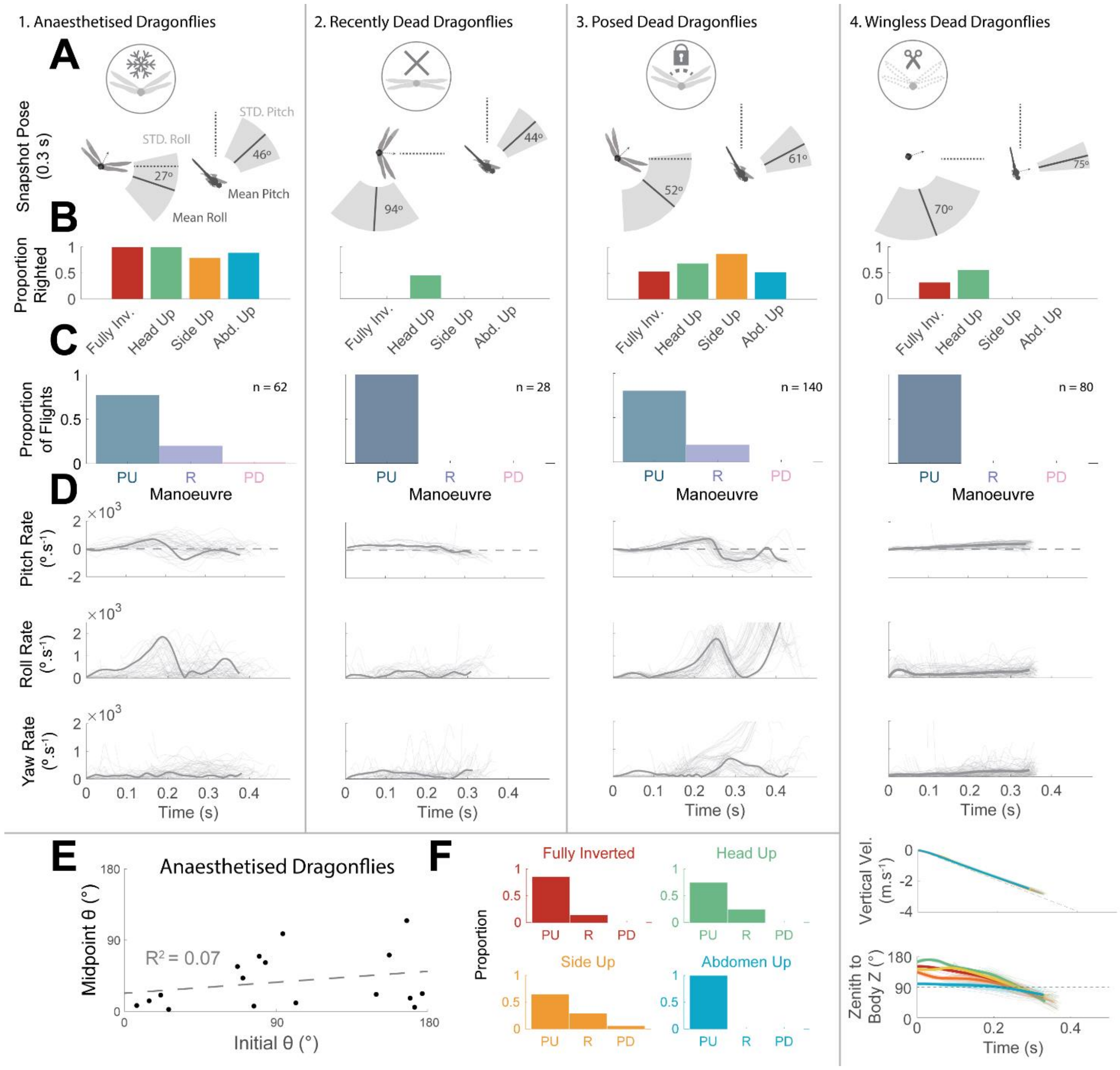

Supplementary Figure 4: Inactive dragonfly righting modes

(A) Mean attitude (pitch and roll) of falling dragonflies at 300 ms into the fall. This time was after most manoeuvring animals had righted. Mean values are given and shaded regions cover ± STD. Dotted lines correspond to 0 values for the pitch and roll vectors. *(B)* Shows the proportion of trials in which the animals' rotated into a correct-side-up attitude (dorsal vector within 45° of the zenith), separated by starting body inclinations. *(C)* The proportion of flights falling into either of three predominant recovery modes: pitch-up, rolling, or pitching-down. *(D)* The pitch- (top), roll- (middle), and yaw-rates (bottom) are shown for each passive drop-type. A single example trace is highlighted in bold for each of the passive conditions. *(E)* The midpoint initial θ versus the initial θ at the start of the fall for those trials in which both points could be resolved. *(F)* The proportion of recovery modes for each initial inclination.

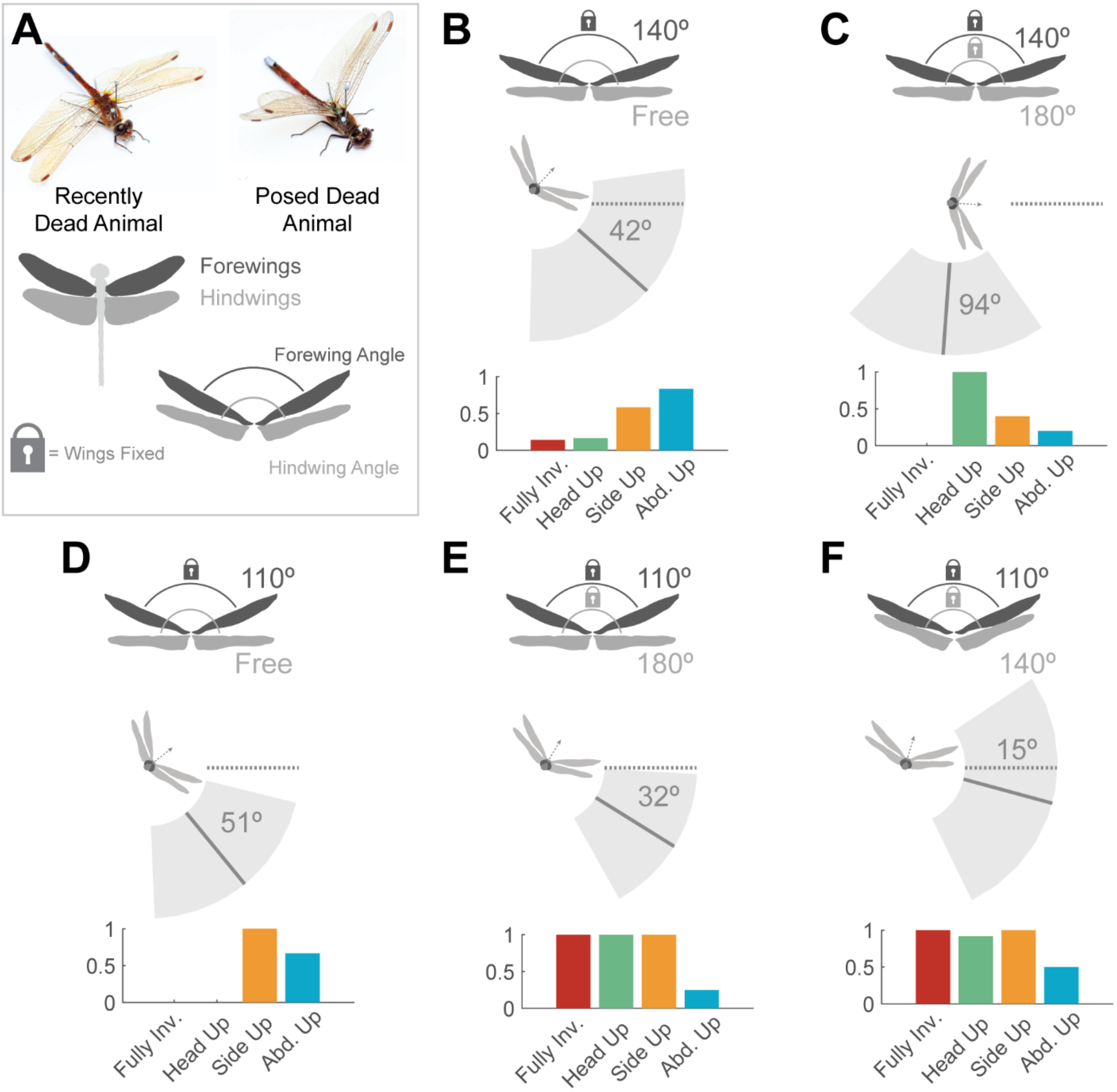

Supplementary Figure 5: Wing pose specific effects

(A) A recently dead animal (left) and posed animal (right) with a schematic of how to read figure icons. Individual panels show the wing pose (top), 300 m snapshot mean of absolute roll (middle), and proportion of trials that achieve righting (bottom) for the following wing poses: *(B)* forewings 140°, hindwings free, *(C)* forewings 140°, hindwings 180°, *(D)* forewings 110°, hindwings free, *(E)* forewings 110°, hindwings 180°, *(F)* forewings 110°, hindwings 140°. Different alternative wing poses produced different results. Fixing both the forewings and hindwings into a dihedral (fore 110° inter-wing angle, hind 140°) produced the best righting response (300 ms snapshot roll of 15.2 ± 48°, n = 48), while the worst recoveries were found with a fixed fore and hind inter-wing angle of 140° and 180° respectively (300 ms snapshot roll of 94 ± 48°, n = 16). The other conditions produced results similar to the population average.

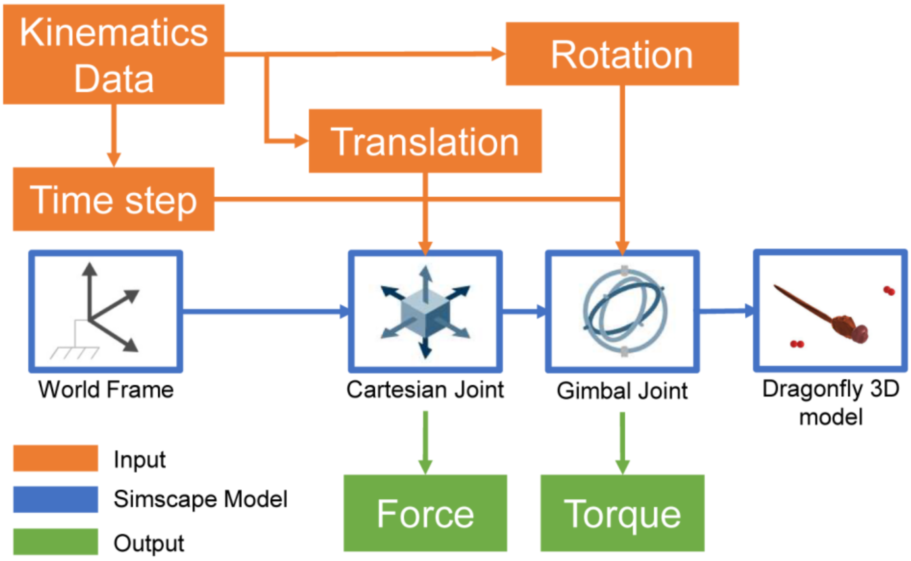

	Thorax	Abdomen	Head	Wings	Total	Total (wingless)
Mass (g)	0.1367	0.0328	0.0305	0.0008	0.232	0.2
Density (kg/m^3)	1280.46	1254.24	652.343			
Volume (mm^3)	106.76	26.15	46.75			
MOI: Ixx (g/mm^2)	0.328	0.009	0.085		1.554	0.431
MOI: Iyy (g/mm^2)	0.769	1.032	0.065		8.412	8.377
MOI: Izz (g/mm^2)	0.689	1.033	0.062		9.385	

Solver	Fixed-step ode1be (Backward Euler)
Jacobian Method	Sparse perturbation
No. of Newton's Iteration	400
Time step	0.0042s
Consistency Tolerance	1.00E-09
Filtering Time Constant	0.001
Gravity	[0,0,0]

Supplementary Figure 6: The inverse dynamic model

(A) An illustration for the inverse dynamic pipeline. A gimbal joint and a cartesian joint were used to reconstruct the translation and rotation of dragonflies' manoeuvre, and estimate the force and torque exerted in each time step. Since we did not simulate aerodynamics, the wings were modelled as point masses 16mm away from wing joints (red spheres). *(B) (Top)* Physics parameter detail for the dragonfly model and *(Bottom)* Simscape solver settings. Since we imposed the body kinematics from experiments (which were driven by gravity), we removed gravity for the simulation and subtracted it from the result to extract aerodynamic effects.

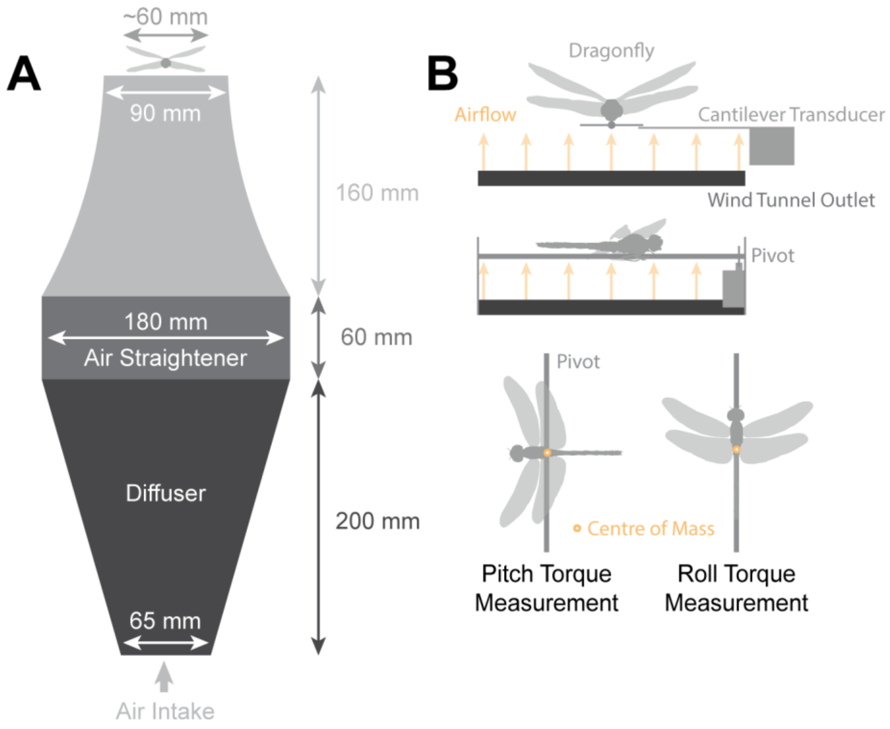

Supplementary Figure 7: The vertical wind tunnel

(A) A schematic of our simple low-speed wind tunnel with dimensions listed. Air straighteners were fully packed by 5 mm diameter straws in the longitudinal direction. An electric ducted fan was fitted at the bottom air intake. *(B)* (*Top*) A schematic diagram for the torque measurement arrangement, showing the pivot and cantilever. (*Bottom*) Alternative alignments of the dragonfly and pivot to measure either pitch or roll torque.
